# EMTscore infers divergent EMT pathways from omics data and enables rapid screening for EMT-associated gene sets

**DOI:** 10.1093/bioinformatics/btag286

**Published:** 2026-05-07

**Authors:** Haimei Wen, Leonidas Bleris, Tian Hong

**Affiliations:** Department of Biological Sciences, The University of Texas at Dallas, Richarson, TX 75080, United States; Department of Bioengineering, The University of Texas at Dallas, Richarson, TX 75080, United States; Department of Biological Sciences, The University of Texas at Dallas, Richarson, TX 75080, United States

## Abstract

**Motivation:**

Quantitative analyses of epithelial-mesenchymal transition (EMT) have been widely used in several areas of biomedical sciences due to its importance in development and cancer progression, but its multi-contextual nature requires standardization and implementation of gene set scoring methods beyond capacities of conventional tools.

**Results:**

We developed EMTscore, a package that provides an efficient implementation of unbiased scoring methods for multiple EMT pathways using individual single-cell or bulk omics data, and the package allows rapid screening for cellular processes correlated with EMT.

**Availability and implementation:**

EMTscore is available from GitHub https://github.com/wenmm/EMTscore under the GNU General Public License, and is uploaded on Zenodo with a DOI 10.5281/zenodo.19487376.

## 1 Introduction

**Figure 1:**
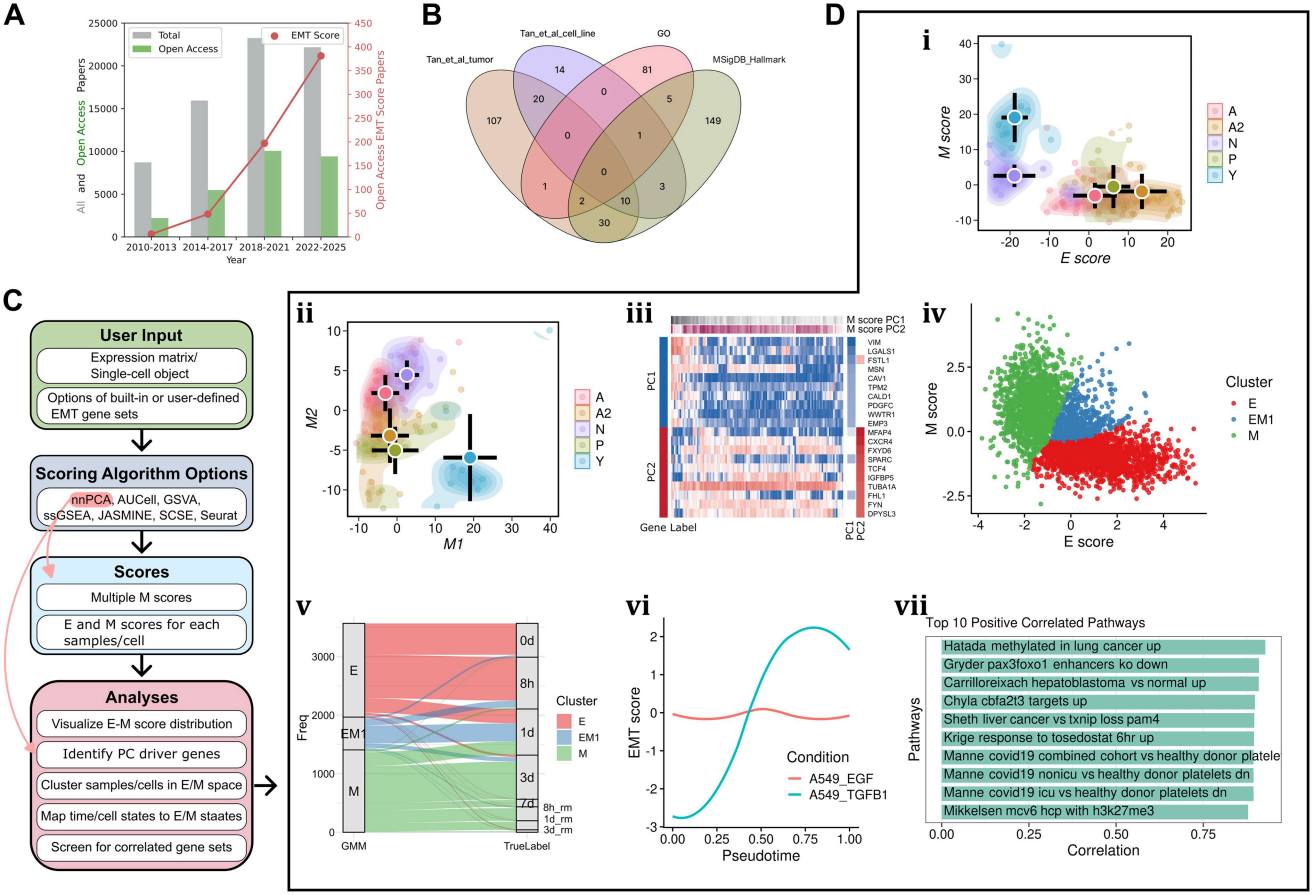
Motivation and overview of EMTscore. (A) EMT-related publication counts with respect to time. Articles with both keywords “epithelial” and “mesenchymal” mentioned in abstracts were extracted from PubMed as of December 31, 2025. Open access, original research articles were identified from this total pool. Among open access articles, papers with keyword “EMT score” were further identified. (B) Venn diagram showing the overlaps of four widely used EMT gene sets. (C) Workflow of EMT score package. (D) Example visualizations from EMScore. (i–iii) Analyses for 120 SCLC bulk RNA-seq samples. (i) nnPCA-based E and M scores. (ii) nnPCA-based M1 and M2 scores. (iii) Expression levels of top genes contributing to M1 and M2 scores. (iv–vii) scRNA-seq data for A549 cells treated with TGF-β or EGF. (iv) GMM based clustering of TGF-β treated cells. (v) Sankey plot showing the mapping of time points and EMT states from GMM clusters. (vi) E and M1 scores as functions of pseudotime for two types of treatments. (vii) Top 10 gene sets whose scores are positively correlated with EMT (M1) scores for TGF-β-treated cells.

Epithelial-mesenchymal transition (EMT) is the cellular process in which epithelial cells with tight cell junction and apical-basal polarity transform into motile mesenchymal cells. EMT is responsible for key steps of development and is activated in diseases progressions such as fibrosis and metastasis (Nakaya and Sheng 2013). Particularly, cancer cells can exploit the gene regulatory network of EMT and the invasiveness of the mesenchymal (M) cells (Thiery et al. 2009). Experimental and theoretical studies have shown that EMT involves multiple intermediate states rather than a binary switch (Lu et al. 2013, Zhang et al. 2014, Hong et al. 2015, Yang et al. 2020), and partial EMT was shown to be crucial for cancer progression (Pastushenko et al. 2018). In addition, divergent transcriptional programs of EMT can be activated in fate changes toward multiple cell subtypes/states even in the same disease (Groves et al. 2022, Youssef et al. 2024). The importance and complexity of EMT gained significant attention from multiple subfields of biomedical sciences. Over the past decade, several thousand EMT-related research articles have been published each year (Fig. 1A).

Due to the multistate nature of EMT and its diverse physiological contexts, EMT cannot be accurately quantified with a small number of genes (Yang et al. 2020). Gene sets analysis is therefore widely used to generate “EMT scores” to assess the degree of EMT in different settings (Fig. 1A) (Tan et al. 2014, Panchy et al. 2022). Scoring EMT with high-throughput experiments, including single-cell RNA-sequencing (scRNA-seq), enabled both discoveries for fundamental understanding of EMT and development of new therapeutic strategies (Elamin et al. 2022, Cassier et al. 2023, Youssef et al. 2024, Groves *et al*. 2023). Meanwhile, to facilitate characterization of EMT pathways, several databases and tools have been developed to date (Zhao et al. 2015, Zhao et al. 2017, Vasaikar et al. 2021). However, these resources do not meet the increasingly complex gene set analyses for EMT. While general gene set enrichment methods are useful for quantifying EMT progression with predefined EMT gene sets, the research in this field has been challenged by several key issues. First, different EMT gene sets have been used in different studies (Liberzon et al. 2011, Tan et al. 2014, Carbon et al. 2021, Panchy et al. 2022), and these gene sets have very limited overlaps (Fig. 1B); the current gene set analysis tools do not allow easy access to diverse gene sets or fast tests with multiple gene sets. Secondly, discoveries and visualization of divergent activations of subsets of EMT genes require *ad hoc* methods with which it is difficult to reproduce key results (Youssef et al. 2024). Finally, there has not been an easy-to-use tool that allows the mapping from standard cell state labels to biologically meaningful EMT states.

We developed an R package named EMTscore that enables easy, simultaneous use of multiple popular EMT gene sets(Taube et al. 2010, Liberzon et al. 2011, Gröger et al. 2012, Tan et al. 2014, Cursons et al. 2015, Du et al. 2016, Foroutan et al. 2017, Cook and Vanderhyden 2020, The Gene Ontology Consortium 2021, Vasaikar et al. 2021, Panchy et al. 2022, Youssef et al. 2024) and scoring algorithms (Sigg and Buhmann 2008, Hänzelmann et al. 2013, Aibar et al. 2017, Pont et al. 2019, Noureen et al. 2022, Hao et al. 2024), as well as in-depth (single-cell) RNA-seq data analyses of multistate EMT and their visualizations with publication-quality plots. We implemented a method based on nonnegative principal component analysis (nnPCA) that enables automatic detection of groups of divergently activated EMT samples and genes in the same dataset. Additionally, the toolbox includes identification of EMT states in low-dimensional functional space and its mapping to user-defined cell clusters. Finally, our package can screen gene sets whose expressions are correlated with EMT progression, and this reveals the relationship between EMT and other biological processes efficiently.

## 2 Software description

### 2.1 Implementation of algorithms for computing EMT scores

To perform scoring of epithelial (E) or mesenchymal (M) gene set activities across transcriptomes (bulk or single-cell samples), EMTscore requires an expression dataset provided as a matrix file, a Seurat object, or a SingleCellExperiment (SCE) object. In addition, EMTscore provides several widely used E/M gene sets which can be replaced by custom gene sets provided by users (Fig. 1C, green).

To enable complex downstream analyses, we implemented multiple scoring algorithms that allow flexibility for choices of popular gene set enrichment methods. A default scoring algorithm based on nonnegative PCA (nnPCA) enables automated detection of multiple EMT programs (Sigg and Buhmann 2008, Panchy et al. 2021) (Fig. 1C, purple). Here, we briefly describe the advantages of the implemented methods. nnPCA is a fast method based on variance of data points (see Supplementary Information, available as supplementary data at *Bioinformatics* online). Its unique advantage of providing multiple axes (e.g. multiple scores) with ranked variances explained makes it suitable for revealing multiple EMT programs in the same dataset. However, nnPCA does not provide a statistical test. In contrast, the widely used single-sample gene set enrichment analysis (ssGSEA) method uses Kolmogorov-Smirnov (KS) test for determining significant enrichment (Barbie et al. 2009), but the method is inefficient to handle large datasets such as scRNA-seq data. Other methods, such as GSVA (Hänzelmann et al. 2013), AUCell (Aibar et al. 2017), SCSE (Pont et al. 2019) and JASMINE (Noureen et al. 2022), are either based on gene ranks or normalized sums of expression (Aibar et al. 2017, Pont et al. 2019, Noureen et al. 2022). They are more efficient than ssGSEA but their statistical rigor varies with context, and they do not allow multiple gene scores from the same dataset.

EMTscore allows users to choose E and/or M gene sets from commonly used sources (Fig. 1B). A default set of E/M genes from Panchy et al. (2022) can be used. This collection combines a data-derived set of EMT genes and additional, widely recognized transcription factors controlling EMT. To enable easy access and application in a unified format, we compiled previously published EMT gene sets into a single GMT file included in this package.

### 2.2 Analysis of multiple EMT transcriptional programs

Upon completion of nnPCA-based scoring, EMTscore extracts multiple leading principal components (PCs) for M scores. Similar to conventional PCA, these PCs are ranked based on their variances explained. For some analyses, one M axis (i.e. the leading PC based on a M gene set) is used, and each sample/cell receives one M score and one E score (Fig. 1D, i). To detect divergent EMT (M) programs, two or more PCs from M scores are used. Typically, the first M PC (M1) and the second M PC (M2) can be used to describe the two directions of M programs driving the variance of the data points (Fig. 1D, ii). Genes whose expressions contribute to M1 and M2 can be extracted based on their rotations (loadings) (Fig. 1D, iii).

EMTscore visualizes the progression of EMT in the space of E-M for conventional EMT scores, and in the space of M1–M2 for divergent EMT programs. Scatter plots with various options are provided for these visualizations (Fig. 1D, i and ii). Note that our package allows user-defined gene sets with an input GMT file, so the capabilities of detecting divergent transcriptional programs can be generalized to study other pathways.

### 2.3 Detection of EMT states with Gaussian Mixture Model

In RNA-seq data, samples are sometimes with experimental labels (e.g. time points). In addition, for scRNA-seq datasets that typically contain a large amount of samples, clustering information is readily available from commonly used packages such as Seurat (Stuart et al. 2019). However, the relationship between these cluster labels and EMT states is often unclear. To provide insights into this question, EMTscore uses Gaussian Mixture Model (GMM) to cluster cells in E-M space and the clusters are interpretable biologically. Next, the package detects the extreme E and M subpopulations as well as hybrid EMT subpopulations relative to the dataset (Fig. 1D, iv). A mapping of the samples’ original labels to EMT states is visualized (Fig. 1D, v) and the numerical distributions are reported. Single-cell level labels, such as pseudotime inferred from other packages, can be analyzed in relation to EMT scores (Fig. 1D, vi).

### 2.4 Screening for cellular processes correlated with EMT

EMT has been shown to have crosstalk with other important cellular processes such as alteration of motility and invasiveness (Jolly and Huang 2014, Wong et al. 2014). It is therefore of interest to search for gene set scores that are correlated and/or anticorrelated with EMT scores. Leveraging the high efficiency of scoring methods such as nnPCA, we implemented a screening function for searching through MSigDB (7561 curated gene sets in C2), and ranking gene sets that are highly correlated/anti-correlated with EMT scores (nnPCA-based EMT scores are M1 scores by default) across samples/cells. EMTscore reports the top positively correlated gene sets and top negatively correlated gene sets separately as lists and bar charts illustrate their Pearson correlation coefficients (e.g. Fig. 1D, vii). A gene set overlapping threshold (default 30%) is used to filter out gene sets with too many overlapping genes with the input gene list.

## 3 An example analysis pipeline

We first analyzed a bulk RNA-seq dataset containing transcriptomes from 120 small cell lung cancer (SCLC) cell lines with various known tumor subtypes (A2, A, N, P, and Y) (Cerami et al. 2012, Groves et al. 2022). To investigate the relationship between the subtypes of SCLC and EMT states, we computed E and M scores with each scoring method, and we found that A2 was an extremely E-like subtype, whereas Y was extremely M-like (Fig. 1D, i and Fig. 1, available as supplementary data at *Bioinformatics* online). The EMT identities for other subtypes were not clear from a single M score. With nnPCA, we found that there was a divergent expression of subsets of M genes between Y and A/N subtypes (Fig. 1D, ii), and that each M direction involves upregulation of a distinct set of M genes. Further analysis with the two leading M PCs showed that M markers such as VIM contributed significantly to PC1 (M1) whereas M2 was supported by other M markers such as CXCR4 (Fig. 1D, iii). Both marker sets are recognized M genes (Mendez et al. 2010, Cheng et al. 2018), but they showed distinct patterns across different SCLC cells. Although VIM is widely used as a key EMT marker due to its role in promoting cell motility (Gilles et al. 1999), it also protects cells from nuclear deformation by increasing nuclear stiffness (Patteson et al. 2019). This latter effect can inhibit cell migration in constricted environments (Patteson et al. 2019). SCLC cells may therefore utilize diverse programs and cell phenotypes to enhance their invasiveness with high adaptiveness.

We next use a scRNA-seq dataset from a time-course experiment profiling EMT induction in 12 911 A549 cells treated with TGF-β (Cook and Vanderhyden 2020). With E-M scoring followed by GMM-based clustering, we found that cells at the hybrid EMT state are mainly distributed at the transition time points of the experiments (Fig. 1D, iv and v), and that the progression of cell states over time was strongly correlated with the downregulation of E genes and upregulation of M genes with the treatment of TGF-β (Fig. 1D, v). This trend was also consistent with the progression of M scores (M1 from nnPCA) over pseudotime (Fig. 1D, vi, blue), which is more widely available from scRNA-seq datasets compared to true time labels. Interestingly, independent analysis of 12 435 A549 cells treated with EGF from the same study did not show such a trend (Fig. 1D, vi). This observation is consistent between nnPCA and other methods (Fig. 2, available as supplementary data at *Bioinformatics* online). Finally, we identified several gene sets whose scores were strongly positively/negatively correlated with M (i.e. EMT) scores (Fig. 1D, vii and Fig. 3, available as supplementary data at *Bioinformatics* online).

In addition to the examples related to cancer progression, we analyzed a scRNA-seq dataset for trunk neural crest (Soldatov et al. 2019), and we found that the E-M scoring from EMTscore not only had reasonable agreement with development trajectories but also gave insights into potential transient expression of some M genes during development (Fig. 4, available as supplementary data at *Bioinformatics* online).

## 4 Conclusions

EMTscore provides flexible, versatile and easy-to-use functionality that allows in-depth analyses with gene set scores for EMT progression and publication-quality visualizations. The package is suitable for both bulk and sing-cell omics datasets. A default nnPCA-based method enables identification of multiple, divergent EMT pathways that are supported by different subsets of M genes, and this method is efficient for screening pathways that are associated with EMT. Future developments of this package will provide more biological insights enabled by combination of EMTscore-based cell states and other computational methods such as intercellular communication inference (Jin et al. 2021, Lopez et al. 2025).

## Supplementary Material

btag286_Supplementary_Data

## Data Availability

EMTscore is available from GitHub https://github.com/wenmm/EMTscore under the GNU General Public License, and is uploaded on Zenodo with a DOI 10.5281/zenodo.19487376.
